# An Account of the Successful Resuscitation of Three Persons from Suspended Animation, by Submersion in the River Ohio, for Twenty or Twenty Five Minutes

**Published:** 1829-01

**Authors:** Richard Povall

**Affiliations:** Philadelphia


WESTERN JOURNAL

OF THE

MEDICAL & PHYSICAL SCIENCES.

JANUARY, 1829.

### ESSAYS AND CASES.

Art. I.—*An account of the successful resuscitation of three persons from suspended animation, by submersion in the river Ohio, for twenty or twenty-five minutes.* By Richard Povall, M. D. of Philadelphia, in a letter to the Editor.

Sir—Your letter bearing date the 21st instant, (December) and in which you express a desire that I should furnish you for publication in the Western Journal of the Medical and Physical Sciences, of which you are the Editor, an account of the means by which I succeeded in restoring three persons of colour from a state of suspended animation by drowning, when the slave boat sunk in the Ohio a few weeks ago, was, by the polite attention of my friend Peter Benson, Esq, of your city, forwarded to me, and was received yesterday. I comply most cheerfully, sir, with your request; but regret that my bad state of health, in combination with other circumstances beyond my control, will prevent me from giving you so full an account of the interesting cases to which you refer as they intrinsically merit. I must therefore, for the present, be content to state, simply, the cases as they occurred, and the means I employed in restoring to animation the sufferers, without entering largely into the inquiry—how it came to pass that the means, simple as they were, and which are not new to the Medical world, should have been so singularly successful in my hands. This I may do, however, at some future day, when circumstances may not be of so discouraging a character, to a satisfactory accomplishment of my views, as they now are.

On an early day of the present month, I was on board the steam boat General Marion, as a passenger, on my way from Wheeling to Cincinnati, and about 8 o'clock, A. M. the steam boat took in tow a slave boat containing 27 slaves of various ages and of both sexes. The slave boat had not been attached to the steam boat one minute, before the former was put into a sinking condition with all hands on board. At this juncture, the greatest confusion ensued on board of both boats:—the crew of the sinking boat were calling upon that of the steam boat, with the most piercing cries, for help!--The jolly boat, belonging to the steam boat, after a short delay was now put off to the succour of the wreck, but it did not get to its relief until seven of the negroes, two adult women and five children, were submersed, and had been kept in that situation at least twenty or twenty-five minutes, by means of a strong board covering erected over the boat for the purpose of protecting the crew from bad weather, and under which, those who suffered suspended animation, and those who finally perished by drowning, were reposing at the time the catastrophe occurred.

There were only five bodies recovered out of seven that suffered submersion; three of whom were perfectly restored to animation and sound health, by the means stated below. One of the other two having been under water more than thirty minutes, the same means were used, but were found inadequate to effect the same salutary end that had been effected in the other cases, though they were persevered in for a long time. The fifth subject did not claim my attention, it having lain under water so long before it was recovered as to preclude all hope of success in restoring it to animation, however skillful and diligent I might have been in the application of my means.

The three successful cases claimed my attention in rapid succession, insomuch, that I found myself working upon each one at short intervals, in less than three or four minutes from the time I commenced upon the first.

The ages of the little sufferers were as follow: the first one handed to me was twenty months—the second fourteen months—and the third three months old. The two first were females, and the last a male. They were taken out of the water, in order of time, exactly as I have recorded their ages, (i. e.) the eldest first, and so on. From the guards of the steam boat I received in my own hands each of the patients and conveyed them immediately to the cabin, which had already been made warm and comfortable by a large fire of some one or two hours standing, before the accident occurred. I next stripped them of their wet clothing, and called into requisition all the dry napkins that were at hand, and with which I wiped them perfectly dry from head to foot. Pillows and blankets were next seized upon, and most valuable means they were in restoring my patients to life and animation. With the head and shoulders a little elevated upon the pillows, and the blankets being warmed, I proceeded to wrap the patients in them, and at the same time to use friction in the gentlest manner, especially over the epigastric region. After a little time, as the signs of returning animation developed themselves, I extended the frictions towards the superior and inferior extremities. These methods having been diligently used for some minutes, I very soon had the satisfaction to perceive that the natural warmth of my patients was fast returning, and that they not only began to breathe freely, but that their powers of deglutition were also restored. At this juncture, I administered, without the loss of a moment, small draughts of warm brandy toddy, a teaspoonful at a time, with the most beneficial results. In this stage of my operations, my patients, each of them, had, spontaneously, copious alvine evacuations, and from that moment they recovered most rapidly. Perceiving they were likely to do well, I now turned them over to the care of nurses, and gave my attention exclusively to the fourth and unsuccessful case, the subject of which was a female, aged about nine or ten years, and as before stated had been under water more than thirty minutes before she was received into the cabin. All the means employed in the three successful cases were diligently applied to this one, in conjunction with others—such as inflating the lungs, stimulating the nostrils, the tongue, and fauces, with such means as were at my command. But all was to no purpose. The vital spark had doubtlessly been entirely extinguished before she was taken from the water.

In three or four hours after the reanimation of my little patients, I visited them on the upper deck of the steam boat, and found them all so much recovered from the effects of their submersion, that a stranger looking on them would not have suspected that any harm had so recently befallen them. About eight hours after this visist, the eldest had some fever, which was very soon subdued by a dose of the sulph. magnesiæ. The other two had no fever whilst I continued to attend them, which was for two consecutive days after the accident.

Considering the great length of time* the three restored patients had been submersed before they were subjected to the operations of the simple means employed in effecting their resuscitation, it would seem, on the first view of the subject, that some superior merit should be ascribed to those means, but in doing that, I am not sure we should be entirely free from error. I am inclined to the opinion, that if these three subjects had been of *adult age*, and *had remained under water the same length of time, the same means of resuscitation might have been used*, and in all probability without such signal success as attended them—because, as the vital principle is known to be

* Most of the respectable authors agree that "when a person has remained above fifteen minutes under water, there can be no considerable hopes of his recovery." The instances recorded by Tissot, of persons having "remained *six hours* under water," and then were "restored to life by the heat of a dung hill," are so exceedingly improbable, that it requires a great stretch of credulity to believe them true.

less tenacious in adult age than it is in extreme youth,* it might not, under the hypothetical circumstances here laid down, be able to preserve itself from total extinction—thus submersion for *twenty-five minutes* might be sufficient to extinguish the vital principle in a subject of adult age, when it would be insufficient to extinguish the vital principle in a very young subject, by reason of its greater tenacity. From these premises I am ready to conclude, that the happy issue of my three successful cases, was more owing to the *superior vital energies of the youthful subjects*, thereby giving to them extraordinary recuperative powers, than to any *superior efficacy to the means employed* in effecting their reanimation.

It may be well to remark in this place, that the youngest subject of the three successful cases of which we have been speaking, was restored to animation first, and with less exertion than either of the other two, although it had been under water a few minutes the longest. The same may be said of the next youngest, compared with the eldest of the three. These facts I consider as in some degree illustrating and establishing the theory which I have nothing more than hinted at in this communication.

* "Children display," says Professor Chapman, in a note in James' Burns, "an uncommon *tenacity of life*, and strength of constitution. They often survive under circumstances which destroy adults. They have often been found living at the breasts of their mothers who had perished by exposure to cold."

